# Multiple barriers for accessing mental health service among women attending shelters for women experiencing intimate partner violence

**DOI:** 10.1192/j.eurpsy.2022.815

**Published:** 2022-09-01

**Authors:** N. Daoud, N. Ali Saleh-Darawshy

**Affiliations:** 1 Ben-Gurion University of the Negev, School Of Public Health, Beer-Sheva, Israel; 2 Bar-Ilan University, School Of Social Work, Ramat Gan, Israel

**Keywords:** Women, intimate partner violence, mental health services, accessibility

## Abstract

**Introduction:**

While women victims of intimate partner violence (IPV) suffer the burden of mental health issues (MHI), they face many challenges accessing mental health services (MHS).

**Objectives:**

We draw on the socioecological model and explore different level barriers for accessing MHS among women experiencing IPV.

**Methods:**

We conducted a qualitative study in 2020-2021 at three levels: policy, practice and women’s experience. This included in-depth interviews with 19 policymakers from the Ministry of Health (MoH) and the Ministry of Social Welfare (MSW); four directors of shelters for women victims of IPV; 35 women (26 Arabs, 9 Jewish) attending shelters for women victims of IPV (age 22-50), and six focus groups with 26 social workers. Participants were asked about the barriers for utilizing MHS.

**Results:**

We identified complex multifaced barriers regarding the accessibility and quality of MHS among women victims of IPV. At the policy level, we identified structural organizational barriers related to the division of responsibilities between the two offices (MoH and MSW). These included lack of collaboration, funding and information transmission and insufficient communication mechanisms. At the practice level, shelters’ directors and social workers raised barriers, most of which were related to divisions in knowledge, terminology, and treatment approaches among mental healthcare providers and social welfare therapists. The women themselves raised issues related to stigma, lack of family support and continuity of MHS.

**Conclusions:**

To improve MHS access, it is crucial to overcome the multiple barriers (individual, family, therapeutic and organizational) that are faced by women who are experiencing IPV.

**Disclosure:**

No significant relationships.

